# Draft Genome Sequence of Pseudomonas sp. Strain LLC-1 (NBRC 111237), Capable of Metabolizing Lignin-Derived Low-Molecular-Weight Compounds

**DOI:** 10.1128/genomeA.00308-18

**Published:** 2018-04-19

**Authors:** Jun Hirose, Naoki Tsuda, Munetoshi Miyatake, Haruhiko Yokoi, Jun Shimodaira

**Affiliations:** a Department of Applied Chemistry, Faculty of Engineering, University of Miyazaki, Miyazaki, Japan; b Bioscience R&D Department, Toa Pharmaceutical Co., Ltd., Tatebayashi, Japan

## Abstract

Pseudomonas sp. strain LLC-1 (NBRC 111237), isolated from soil, metabolizes lignin-derived low-molecular-weight compounds and utilizes vanillin and vanillic acid as its sole sources of carbon. Here, we report the draft genome sequence of Pseudomonas sp. strain LLC-1.

## GENOME ANNOUNCEMENT

Due to a recalcitrant nature, only limited groups of organisms, such as white-rot fungi, can degrade lignin. However, microorganisms capable of metabolizing lignin-derived low-molecular-weight compounds (LLCs) are ubiquitous in the natural environment, and their primary metabolism can be used to generate useful bioproducts from lignin (1). Vanillin, vanillic acid, and syringaldehyde are major components of LLCs. We isolated a Gram-negative aerobic bacterial strain, Pseudomonas sp. strain LLC-1, by using an enrichment culture in a medium containing LLCs and inorganic salts (2). This bacterial strain can utilize vanillin and vanillic acid as its sole sources of carbon and cometabolizes syringaldehyde, *o*-vanillin, and isovanillin. Here, we present the draft genome sequence of Pseudomonas sp. strain LLC-1.

The draft genome sequence of strain LLC-1 was determined by a combined method using the MiSeq system (Illumina) with paired-end runs and the 454 GS FLX+ system (Roche). A hybrid assembly of the reads obtained by the two sequencing methods was performed using Newbler version 2.6 (Roche). The assembled genome is composed of 42 contigs (>537 bp) totaling 5,946,122 bp, with a G+C content of 62.4%. The *N*_50_ contig size and the largest contig size are 322,774 bp and 480,886 bp, respectively.

The genome annotations were performed using the NCBI Prokaryotic Genome Annotation Pipeline (PGAP) (3) and the Rapid Annotations using Subsystems Technology (RAST) server version 2.0 (4). PGAP predicted 5,208 coding DNA sequences (CDSs) and 66 tRNA sequences. RAST predicted 5,275 CDSs and 64 tRNA sequences. The coding sequences were classified by RAST into 4,334 subsystems, of which the systems for the metabolism of amino acid derivatives (*n* = 752 CDSs), carbohydrates (*n* = 475), cofactors, vitamins, prosthetic groups, and pigments (*n* = 344), and protein metabolism (*n* = 287) were the most abundant. Comparison of the genome sequences available in the RAST data sets revealed that P. putida GB-1 (5), with a score of 513, is the closest neighbor of strain LLC-1, followed by P. putida F-1 (6), with a score of 504. We are identifying the genes coding for vanillin metabolism from the draft genome sequence of strain LLC-1. An interesting feature of strain LLC-1 (2) is that it can metabolize vanillin and vanillic acid without reported inducer compounds, such as eugenol (7) or feruloyl-coenzyme A (CoA) (8). The whole-genome sequence of strain LLC-1 will enable the identification of genes coding enzymes metabolizing LLCs and will help advance our understanding of its unique vanillin metabolism.

### Accession number(s).

The draft genome sequence of Pseudomonas sp. strain LLC-1 has been deposited in GenBank/ENA/DDBJ under the accession number LUVY00000000.
